# Screening *in silico* predicted remotely acting *NF1* gene regulatory elements for mutations in patients with neurofibromatosis type 1

**DOI:** 10.1186/1479-7364-7-18

**Published:** 2013-08-15

**Authors:** Stephen E Hamby, Pablo Reviriego, David N Cooper, Meena Upadhyaya, Nadia Chuzhanova

**Affiliations:** 1 School of Science and Technology, Nottingham Trent University, Clifton Lane, Nottingham NG11 8NS, UK; 2 Institute of Medical Genetics, School of Medicine, Cardiff University, Heath Park, Cardiff CF14 4XN, UK; 3 Current address: Department of Cardiovascular Sciences, University of Leicester, Glenfield Hospital, Leicester LE3 9QP, UK

**Keywords:** NF1, Neurofibromatosis type 1, Gene mutation, Hi-C data, Histone H3K27ac, Enhancer

## Abstract

Neurofibromatosis type 1 (NF1), a neuroectodermal disorder, is caused by germline mutations in the *NF1* gene. NF1 affects approximately 1/3,000 individuals worldwide, with about 50% of cases representing *de novo* mutations. Although the *NF1* gene was identified in 1990, the underlying gene mutations still remain undetected in a small but obdurate minority of NF1 patients. We postulated that in these patients, hitherto undetected pathogenic mutations might occur in regulatory elements far upstream of the *NF1* gene. In an attempt to identify such remotely acting regulatory elements, we reasoned that some of them might reside within DNA sequences that (1) have the potential to interact at distance with the *NF1* gene and (2) lie within a histone H3K27ac-enriched region, a characteristic of active enhancers. Combining Hi-C data, obtained by means of the chromosome conformation capture technique, with data on the location and level of histone H3K27ac enrichment upstream of the *NF1* gene, we predicted *in silico* the presence of two remotely acting regulatory regions, located, respectively, approximately 600 kb and approximately 42 kb upstream of the *NF1* gene. These regions were then sequenced in 47 NF1 patients in whom no mutations had been found in either the *NF1* or *SPRED1* gene regions. Five patients were found to harbour DNA sequence variants in the distal H3K27ac-enriched region. Although these variants are of uncertain pathological significance and still remain to be functionally characterized, this approach promises to be of general utility for the detection of mutations underlying other inherited disorders that may be caused by mutations in remotely acting regulatory elements.

## Background

Germline mutations of the *NF1* gene cause the tumour predisposition syndrome, neurofibromatosis type 1 (NF1), which affects 1/3,000–4,000 individuals worldwide. The *NF1* gene, spanning 283 kb on chromosome 17q11.2, contains 61 exons that give rise to a 12-kb mRNA transcript encoding neurofibromin [1,2]. The 327-kDa (2,818-amino acid) protein product, neurofibromin, is expressed in most tissues.

Neurofibromin is a tumour suppressor protein, reflecting its role as a key negative regulator of the cellular Ras signalling pathway (reviewed by Bennett et al. [3]). More specifically, it is a Ras-specific GTPase-activating protein (GAP), with strong structural and sequence homologies to the GAP superprotein family [4]. It functions by downregulating Ras, thereby resulting in an overall reduction in cellular mitogenic signalling via the Ras pathway. Thus, any *NF1* gene mutation that serves to inactivate neurofibromin function may be expected to increase cellular levels of active Ras-GTP significantly, leading to uncontrolled cell growth and, potentially, tumorigenesis [5]. Germline mutations in the *SPRED1* gene have previously been detected in individuals with clinical features overlapping those of NF1 patients [6].

Consistent with Knudson's two-hit hypothesis, NF1 patients harbouring a heterozygous germline *NF1* mutation develop neurofibromas upon somatic mutation of the second wild-type *NF1* allele. The somatic loss of the second *NF1* allele in the progenitor cell (either the Schwann cell or its precursor), combined with haploinsufficiency in a variety of supporting cells [2,7], is then required for tumour formation. The mutation rate at the *NF1* locus is one of the highest reported in any human disorder [8]; almost 50% of all NF1 patients exhibit a *de novo NF1* mutation. The *NF1* mutational spectrum is shaped in large part, and often in remarkably predictable ways, by the local DNA sequence environment [9]. Nearly 1,300 different inherited mutations of the *NF1* gene have been reported as a cause of NF1; these vary in size from deletions spanning more than a megabase to subtle single base-pair substitutions that alter an encoded amino acid or the function of a splice junction [9]. In classical NF1 patients, *NF1* gene mutations are detectable in 50% to 95% of individuals depending upon the mutation detection techniques employed and the source of tissue used for analysis [2,10]. In our own study, the underlying pathogenic *NF1* mutation was detected in approximately 92% of NF1 patients despite sequencing the exons, splice junctions, untranslated regions and proximal promoter region of the *NF1* gene, and screening for gross gene deletions and rearrangements as well as subtle lesions [11-13]. The undetected mutations in such patients could in principle reside within a remote regulatory element at some distance from the *NF1* gene.

In the human genome, not all regulatory elements occur immediately 5′ to the genes that they regulate; indeed, some are located at considerable distances from their cognate genes. A variety of micro-lesions causing human inherited disease have been found to occur >10 kb 5′ upstream of the corresponding disease genes [14]. These include a total of eight mutations within a 1-kb region (termed the long-range or limb-specific enhancer, ZRS) approximately 979 kb 5′ to the transcription initiation site of the sonic hedgehog (*SHH*) gene [15]. Far upstream polymorphic variants that influence gene expression and impact on disease have also been documented. Thus, for example, the C>T functional single nucleotide polymorphism (SNP) 14.5 kb upstream of the interferon regulatory factor 6 (*IRF6*) gene, which is associated with cleft palate, alters the binding of transcription factor AP-2α [16]. Similarly, a T>C functional SNP approximately 6 kb upstream of the α-globin-like *HBM* gene serves to create a binding site for the erythroid-specific transcription factor GATA1 and interferes with the activation of the downstream α-globin genes [17]. Finally, a T>G functional SNP approximately 335 kb upstream of the *MYC* gene increases the risk of colorectal and prostate cancer by altering the binding strength of transcription factors TCF4 and/or TCF7L2 within a transcriptional enhancer [18]. On the basis of these findings in a number of different genes, we postulated that remotely acting regulatory elements might be present far upstream of the *NF1* gene and that these could be mutated in NF1 patients in whom no pathogenic mutations had been detected.

The *NF1* gene contains a TATA-less promoter within a classic CpG island that extends from the proximal promoter into exon 1, and a 454-bp 5′ untranslated region [19]. The CpG island is normally unmethylated, and the gene is actively transcribed in all tissues so far examined. Despite the high degree of conservation of the proximal regulatory sequences in rodent orthologs [20,21], the distal upstream sequence, potentially harbouring additional regulatory elements, diverges quite significantly between different mammalian species.

Histone H3K27ac serves to distinguish active enhancers from inactive ones [22]. Information on various functional elements, including regions enriched in histone H3K27ac, encoded by the human genome, have recently become available through the ENCODE project resources (ENCODE Project Consortium 2011 [23]; http://genome.ucsc.edu/index.html), a comprehensive collection of experimental data produced using biochemical assays mapped to human genome data. Using a variety of high-throughput techniques for identifying features characteristic to enhancers (e.g. specific histone modifications), up to 1.4 million putative enhancers have been identified in the human genome [23,24].

It has long been hypothesized that communication between widely spaced genomic elements is facilitated by the spatial organization of chromosomes that brings genes and their cognate regulatory elements into close spatial proximity [25]. The development of ‘chromosome conformation capture’ techniques has led to the discovery of many long-range interactions, both intra- and inter-chromosomal (reviewed in [26]). Recently, Lieberman-Aiden et al. [27] employed a novel approach, based on chromosomal conformation capture techniques, and termed Hi-C, to probe the 3D architecture of the entire human genome by coupling proximity-based ligation with massively parallel sequencing to produce a catalogue of approximately 8.4 million inter- and intra-chromosomal interacting fragments. Approximately 6.7 million of these fragments were found to be of long range (>20 kb apart). A genome-wide matrix of DNA-DNA interactions (also known as a contact map), created by dividing the genome into 1-Mb regions and counting the number of interactions between these 1-Mb regions [27], was subsequently analyzed by Yaffe and Tanay [28] to eliminate various biases in experimental procedure.

In this study, we postulated that remotely acting regulatory elements might occur within DNA fragments that interact with the DNA fragment containing the *NF1* gene, and further that these elements might be located within an H3K27ac-enriched region. We first combined long-range DNA-DNA interaction data and ENCODE resources to predict *in silico* the location of novel remotely acting regulatory regions upstream of the *NF1* gene. Finally, we screened these regions for mutation in those NF1 patients in whom no mutations had been found in either the *NF1* or *SPRED1* genes. Germline mutations in the *SPRED1* gene have previously been detected in individuals with clinical features overlapping those of NF1 patients [6].

## Results and discussion

**Figure 1:**
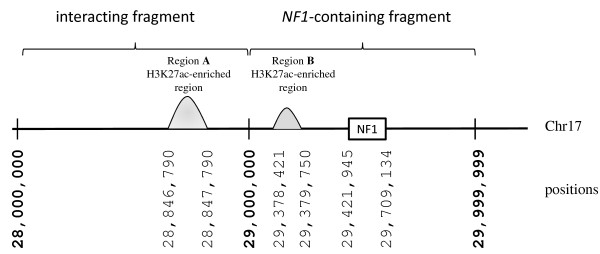
**Schematic of the occurrence and level of H3K27ac enrichment within interacting and NF1**-**containing DNA fragments.** The level of enrichment is shown by the *height of the curves* (not to scale).

The Hi-C data indicated that a 1-Mb fragment of chromosome 17, starting at position 29,000,000 and ending at position 29,999,999, termed the *NF1*-containing fragment since it contained the entire *NF1* gene (positions 29,421,945–29,709,134), was found to interact most strongly with the adjacent 1-Mb fragment on chromosome 17 (703 and 255 intra-chromosomal interactions with the *NF1*-containing fragment recorded in *Hind*III*-* and *Nco*I*-*maps, respectively) which occupies positions 28,000,000–28,999,999 upstream of the *NF1*-containing fragment (Figure 1). The ENCODE data indicated the presence of an H3K27ac-enriched region somewhere between positions 28,846,790 and 28,847,790 in the interacting fragment (region A in Figure 1). This region is distal (approximately 600 kb upstream) to the *NF1* gene. Another peak in H3K27ac enrichment was observed within the *NF1*-containing fragment (region B between positions 29,378,421 and 29,379,750) which is proximal (approximately 42 kb) to the *NF1* gene. The level of enrichment was higher in the distal region A than in the proximal region B. ENCODE data also revealed the presence in region A of three overlapping binding sites for the transcription factors, *p300* (positions 28,846,840–28,847,158), *c-Fos* (28,846,815–28,847,158) and *c-Jun* (28,846,819–28,847,094), all of which were identified by *in vitro* ChIP-seq assay (http://genome.ucsc.edu/index.html).

Two sets of primers (Table 1) were designed using Primer3 software [29]. These were used for the polymerase chain reaction (PCR) amplification and sequencing of the two regions of interest: region A (positions 28,846,790–28,847,790) and region B (positions 29,378,421–29,379,750), from the genomic DNA of 47 NF1 patients as described in the ‘Methods’ section.

**Table 1:** **List of primers used to amplify and sequence DNA regions A** (**28**,**846**,**790–28,847,790) and B (29,378,421–29,379,750)**

**Primer ID**	**Sequence (5′>3′)**
chr17_28846841_1F	GCAAACATAGGAAGTGGCTTAC
chr17_28846841_2F	GAGGGGAGAAGTGTCTGTGTG
chr17_28846841_3F	TTTTCCCATTTAGAGATAAGTCAGTG
chr17_28846841_1R	TTCTGAGGTGCAGTCTTTTAGG
chr17_28846841_2R	GGTTACAACAAACAAGTGGAGAATG
chr17_28846841_3R	TACTTAGGGTGACAAGGCAAG
chr17_29387421_1F	TCTCCCTATGATGTCCAGGC
chr17_29387421_2F	TGCAGCAATAGGTGACTAAAACAC
chr17_29387421_3F	AGGTCCAAATGCAGGTGTG
chr17_29387421_4F	TCTGGAAGCTCTGCTTGG
chr17_29387421_1R	AATTGTTGGCATGAAAACTGG
chr17_29387421_2R	AGATGTGTACCCTGCGGAAG
chr17_29387421_3R	ACGAAGAGATGTGGTTTCCC
chr17_29387421_4R	AACATCCCCTTCCAATTTCC

Three sequence variants were identified in the distal region, the region that corresponds to the highest level of histone H3K27ac enrichment. DNA sequencing revealed the presence of a heterozygous novel C>T variant at position 28,846,793 in two NF1 patients. This variant is not present in the dbSNP database (http://www.ncbi.nlm.nih.gov/sites/entrez?db=snp). Two other variants, rs78190160 (position 28,847,605) and rs71372224 (position 28,846,883), were detected each in three different patients. Both of these variants are listed in dbSNP as being of unknown clinical significance; minor allele frequency/minor allele count based on 1000 Genomes Project data was 0.007/16, indicating that they are rare variants. Unfortunately, no linkage disequilibrium data (HapMap Project; http://hapmap.ncbi.nlm.nih.gov/) were available for this region. The rs71372224 SNP was however found to occur within the three overlapping binding sites for transcription factors *p300*, *c-Fos* and *c-Jun.* Recent studies [30] have shown that enhancer elements have a high propensity to be bound by *p300*, an enhancer-binding transcription factor. One may therefore speculate that a mutation in this putative enhancer element could influence the expression of the *NF1* gene in a spatially specific manner. No variants were found in the H3K27ac-enriched region (region B) lying proximal to the *NF1* gene region and corresponding to the lower level of H3K27ac enrichment.

## Conclusions

This pilot study has shown that a combination of long-range DNA sequence interaction data and information about the location of functional elements available from ENCODE allows us to predict the possible location of novel regulatory regions that may act at distance from the genes whose expression they might influence. Mutations in these regions may in turn modulate the expression of their cognate target gene(s) that are spatially close to these regions. Although the functional ascertainment of these variants far upstream of the *NF1* gene has not yet been performed, the type of *in silico* analysis described here served to guide the mutation screening procedure, allowing us to detect sequence variants in five unrelated patients in whom no pathogenic mutations had previously been found in either the *NF1* gene or the *SPRED1* gene [6]. Even if eventually none of the detected sequence variants turn out to be of pathogenic significance, the Hi-C data indicate that several other 1-Mb fragments have a high number of interactions with the *NF1*-containing fragment and could potentially harbour remotely acting regulatory elements. Among them are two 1-Mb fragments occurring 1 Mb (positions 31,000,000–31,999,999) and 2 Mb (positions 32,000,000–32,999,999) downstream of the *NF1*-containing fragment that have, respectively, 699 and 150 intra-chromosomal interactions with the *NF1*-containing fragment in the *Nco*I-map and, respectively, 222 and 59 interactions in the *Hind*III-map. Another, albeit weaker, inter-chromosomal interaction (21 interactions in the *Nco*I-map) was recorded between the *NF1*-containing fragment and a 1-Mb fragment on chromosome 1 (positions 1–1,999,999). Hence, applicability of this ap-proach is not only confined to *cis*-acting regulatory elements but could also be employed with *trans*-acting regulatory elements. The use of additional information that recently became available through the ENCODE resources, i.e. on the occurrence of CTCF binding factors linked to a range of gene regulatory functions including enhancer-blocking activity of insulator elements; DNase I hypersensitive sites characterizing open chromatin conformations, etc., may help to improve the *in silico* prediction of remotely acting regulatory elements. This approach therefore promises to be of potential utility in investigating those other disorders in which mutations could occur in remotely acting regulatory elements.

## Methods

### Patient samples

A sample of 47 unrelated NF1 patients (out of a total of 624 NF1 patients studied in our diagnostic centre), who tested negative for *NF1* (and *SPRED1*) point mutations and intra-genic insertions/deletions, was selected for this study. This project was approved by the local Ethics committee. Informed consent for sample collection was obtained according to the protocols approved by this committee.

### Bioinformatics analysis

Long-range chromosomal DNA sequence interaction data were downloaded from http://compgenomics.weizmann.ac.il/tanay/?page_id=283. These data comprised the Hi-C contact maps [27], normalized to eliminate various biases associated with the experimental procedure [28]. Two matrices of interactions between 1-Mb fragments, each combining the results obtained using, respectively, the restriction enzymes *Hind*III and *Nco*I in the experimental procedures, were available. Henceforth, these contact maps will be termed, respectively, the *Hind*III- and *Nco*I-maps. Information on the genome-wide occurrence of one particular functional element, the histone H3K27ac-enriched region, was downloaded from the ENCODE database available at http://genome.ucsc.edu/index.html.

The *NF1* gene, spanning positions 29,421,945–29,709,134 from pter of chromosome 17 (NCBI build 37, UCSC HG18 and Ensembl GRCh37), occurs almost in the middle of a 1-Mb DNA fragment that starts at position 29,000,000 and ends at position 29,999,999. This 1-Mb fragment will henceforth be termed the *NF1-*containing fragment. Two of the contact maps described above were searched for DNA fragments, whether occurring on the same chromosome or on different chromosomes that had the most interactions with the *NF1*-containing fragment.

Data on the occurrence and level of H3K27ac enrichment (as measured by the ChIP-seq assay) within the 1-Mb fragment that had the strongest interaction with the *NF1*-containing fragment were downloaded from the ENCODE database. H3K27ac data were also downloaded for the *NF1*-containing fragment. For each of these two 1-Mb fragments (i.e. the fragment that had the strongest interaction with the *NF1*-containing fragment and the *NF1*-containing fragment itself), relatively short regions, corresponding to the highest level of H3K27ac enrichment, termed regions A and B, respectively, were selected for DNA sequencing in 47 NF1 patients in whom no pathogenic mutations had been found by screening both the *NF1* and *SPRED1* genes.

### PCR and sequencing

Two sets of primers for regions A and B, selected as described above, were designed using Primer3 software [29] and purchased from Eurogentec (Southampton, UK). Specificity was confirmed by BLAST analysis.

The genomic DNA isolated from the peripheral blood cells of the NF1 patients was subjected to PCR and Sanger sequencing as described [31]. PCR reaction mixtures of 25 μl contained 5 μl of genomic DNA (6 ng/μl), 5 pmol each of the forward and reverse primers, 0.25 U AmpliTaq Gold polymerase, 3 mM MgCl_2_, PCR buffer and 0.125 mM each dNTP. PCR cycling conditions were 95°C for 10 min followed by 40 cycles of 95°C × 30 s, 60°C × 30 s, 72°C × 30 s with a final extension step of 72°C for 10 min. PCR fragments were confirmed by gel electrophoresis in 1.5% agarose gels and visualized with ethidium bromide staining. The sequencing of the PCR products was performed with BigDye Terminator v3.1 Cycle Sequencing Kits and run on an ABI 3730 genetic analyzer. The obtained sequences were analyzed using Sequencher software version 4.7 (Gene Codes Corp., Ann Arbor, MI, USA). All reagents were purchased from Life Technologies Ltd, Paisley, UK.

## Competing interests

The authors declare that they have no competing interests.

## Authors’ contributions

NC conceived the approach. SEH performed the *in silico* analysis. MU and PR performed the PCR and sequencing. NC, PR and DNC interpreted the results. NC drafted the manuscript. All authors participated in the preparation of the manuscript, and read and approved the final manuscript.
